# Public knowledge and perception about antimicrobials and antimicrobial resistance in Japan: A national questionnaire survey in 2017

**DOI:** 10.1371/journal.pone.0207017

**Published:** 2018-11-05

**Authors:** Kazuhiro Kamata, Yasuharu Tokuda, Yoshiaki Gu, Norio Ohmagari, Katsunori Yanagihara

**Affiliations:** 1 Disease Control and Prevention Center, National Center for Global Health and Medicine Hospital, Tokyo, Japan; 2 Emerging and Re-Emerging Infectious Diseases Unit, National Institute for Infectious Diseases “Lazzaro Spallanzani”, Rome, Italy; 3 Muribushi Project for Okinawa Residency Programs, Okinawa, Japan; 4 AMR Clinical Reference Center, National Center for Global Health and Medicine Hospital, Tokyo, Japan; 5 Department of Laboratory Medicine, Nagasaki University Graduate School of Biomedical Sciences, Nagasaki, Japan; University of Campania, ITALY

## Abstract

**Background:**

Antimicrobial resistance (AMR) is a threat to global health. To increase public awareness about AMR and encourage the prudent use of antimicrobials is one of the goals of the National Action Plan in Japan.

**Methods:**

A nationwide online cross-sectional survey was conducted to evaluate the existing knowledge and perception of AMR in Japan, based on the Antimicrobial Resistance Eurobarometer Survey. Participants included Japanese adults aged 20–69 years, who were not medical professionals.

**Results:**

Among a total of 3,390 participants, about half had taken antibiotics over the past 12 months, and majority of them obtained the antimicrobials from healthcare institutions for the common cold. While 11.7% of the participants kept leftover antibiotics, 23.6% of them have adjusted doses by themselves. About 10% of the participants have requested antibiotics from their doctors, and nearly 30% of them preferred doctors who prescribed antibiotics when had a cold. The common informational sources were TV news and newspapers, and more than 40% of the participants reported receiving some information over the past year. However, approximately 80% of the participants did not know that antibiotics do not kill viruses and that antibiotics are ineffective against cold and flu.

**Conclusion:**

Not many Japanese have adequate information about antimicrobials and AMR, and many have taken antimicrobials inappropriately. Greater educational interventions are, therefore, necessary to increase public awareness and develop effective countermeasures against AMR in Japan.

## Introduction

Antimicrobial resistance (AMR) is the ability of microbes to resist the cytotoxic effects of drugs [1]. Over several decades, microbes causing common or severe infections have developed drug resistance to varying degrees [2], making AMR a global health concern. The United Kingdom Review on Antimicrobial Resistance estimated that, if the resistance was maintained at the current level, by 2050, deaths due to AMR would reach 10 million per year [3].

It is recommended that healthcare professionals use antimicrobials prudently as well as that the general public people obtain appropriate information regarding AMR, to prevent its emergence and spread. In Europe and the United States, public education campaigns to promote awareness of AMR have been carried out, and the effects of these campaigns are evaluated through surveys using questionnaires. Accordingly, these countries are able to assess the educational contents of their campaigns and develop strategies to improve them [4–11]. In Japan, on the other hand, only few efforts have been made to evaluate the public awareness and perception of AMR, one study showed there were some people who do not have adequate knowledge and perception about effects and use of antimicrobials [12]. Thus, we aimed to conduct a nationwide online survey to evaluate the existing knowledge and beliefs among the Japanese public regarding antimicrobials as a first step to promote awareness of AMR.

## Methods

A web-based questionnaire was developed and used to collect responses anonymously. When participants first visited the website for the survey, the policy for using the collected data and protection of personal information was displayed. Only those who agreed for the informed consent with the policy were allowed to answer the questionnaire. The approval of the Ethics Committee for this study was not required for the Ministry of Health, Labour and Welfare, the Government of Japan, because of the anonymous question survey.

### Study participants

Our nationwide cross-sectional survey was designed and conducted online in March 2017 using a nationally representative sample. From a nationwide public panel of 7.6 million people registered with the INTAGE Corporation, a research company, the survey participants were selected from Japanese adults aged 20–69 years. People aged 70 years or older were excluded because of the potential difficulties in responding to the online survey and the same age criteria as the European study (20–69 years) for adult participants was preferred for further comparison of results between Europe and Japan [4]. Additionally, the participants were selected to reflect the distribution of the population (national population census of Japan in 2015) with regards to sex, age, place of residence (prefecture) and population size. Our study sample calculation was based on that of the previous European study [4]. In Germany of that survey, the sample size was 1,563 and the population size (71,283,580 in 2016) of Germany was about half as that (111,207,935 in 2015) of Japan, although national land sizes of Germany and Japan are similar. Thus, we estimated the required sample size of 3,126 (1,563 multiplied by 2), which was two times as the sample size of German people in the European study.

### Data collection

We collected data on sex, age (20–24, 25–39, 40–54 and 55–69 years), education level (lower secondary, secondary, junior college or technical college, university and graduate school), employment status (employed, students, househusband or housewife, unemployed or retired) and hometown size (metropolitan; wards or ordinance-designated cities, mid-sized city; cities with 100,000 or more inhabitants, small city; cities with less 100,000 inhabitants, rural areas; towns or villages). In Japanese healthcare system, all people are basically required to have some types of health insurance and they have free access to visit clinics or hospitals as well as generalist physicians or specialists.

The questionnaire was developed following a consensus among the investigators based on the questionnaires of the European Commission: Special Eurobarometer 445 (Antimicrobial Resistance 2016) and of other relevant studies [4, 7, 13–15]. It included four categories: Use of antibiotics, Knowledge about antibiotics and AMR, Antibiotic information in Japan, and Behaviour and perception at the time of medical visit.

The items of this questionnaire were classified into 4 parts as follows:

1. Use of antibiotics
  - Antibiotics use during the last year
  - Reasons for taking antibiotics
  - Routes of obtaining antibiotics
  - Storage and usage of left-over antibiotics
  - Have you stopped antibiotics or adjusted (made changes to) the dose or the number of antibiotics during the treatment course by yourself?
2. Knowledge about antibiotics and AMR
  - Do antibiotics kill viruses?
  - Are antibiotics effective against cold and flu?
  - Does unnecessary use of antibiotics make them ineffective?
  - Does excessive use of antibiotics result in side-effects such as diarrhoea?
  - Have you heard of the term ‘Antimicrobial Resistance’?
  - What is ‘Antimicrobial Resistance’?
  - What is a cause of ‘Antimicrobial Resistance’?
3. Antibiotic information in Japan
  - Taking information
  - Routes of obtaining information
  - Impact of the antibiotic information on perception and behaviour
  - The most trustworthy sources of information
4. Behaviour and perception at the time of the medical visit
  - Have you ever asked medical doctors to prescribe antibiotics?
  - Do you think medical doctors who prescribe antibiotics for cold are good?
  - Individual responsibility in preserving the effectiveness of Antibiotics

The first part of the questionnaire covered the use of antibiotics. In the second part of the questionnaire about knowledge about antibiotics and AMR, participants were asked four questions about antibiotics and four questions about AMR. For each of the eight questions, responses were true, false, or no idea for statements, including that participants were asked whether antibiotics could kill viruses. Regarding questions for AMR, respondents were asked whether they had ever heard the term ‘antimicrobial resistance,’ Participants were asked two questions: (a) is it true or false that AMR describes humans becoming immune to antibiotics and (b) is it true or false that AMR describes how bacteria avoid being killed by antibiotics. Next, in the third part, we asked participants about how to obtain antibiotic information. The fourth part of the questionnaire dealt with behaviour and perception of participants about antibiotic prescription and if they had ever asked medical doctors to prescribe antibiotics at the time of medical visit. Descriptive statistics, percentages for proportions, were used for the analyses. Data from the Eurobarometer were used as benchmark data [4].

## Results

### Characteristics of participants

We received 3,390 responses over a 5-day recruitment period in March 2017. Table 1 summarizes the sociodemographic characteristics of the participants. All age groups and hometown sizes are well represented in the 2015 national census. The proportion of the total Japanese population that graduated from a university or graduate school (19.9%) was lower than what we found in this study, while the proportion of unemployed and/or retired population (21.7%) was higher than that seen in this study [16, 17].

**Table 1:** Characteristics of participants (N = 3,390).

Characteristics	Study Participants, N (%)	Japanese Population*, N (%)
Sex		
Man	1,736(51.2)	61,841,738(48.7)
Woman	1,654(48.8)	65,253,007(51.3)
Age		
20–24 Years	264(7.8)	5,968,127(7.5)
25–39 Years	953(28.1)	22,016,647(27.5)
40–54 Years	1,109(32.7)	26,325,318(32.9)
55–69 Years	1,064(31.4)	25,614,123(32.0)
Education		
Junior High School	111(3.4)	16,756,162(18.8)
High School	1,265(38.2)	41,400,268(46.5)
College	774(23.4)	13,187,048(14.8)
University/Graduate School	1,158(35.0)	17,716,535(19.9)
Employment		
Employed	2,502(73.8)	58,919,036(57.5)
Housemaker/Housewife	493(14.5)	15,206,558(14.8)
Students	102(3.0)	6,196,077(6.0)
Unemployed/Retired	293(8.6)	22,224,112(21.7)
City Size		
Metropolitan	1014(29.9)	36769964(28.9)
Midsize City	1439(42.4)	51969410(40.9)
Small City	683(20.1)	27397858(21.6)
Rural	254(7.5)	10957513(8.6)
**Total**	**3390**	**127,094,745**

* As of National Census of Japan 2015

### Use of antibiotics (Table 2)

**Table 2:** Use, storage and adjustment of antibiotics.

	Antibiotics Use, N (%)	Storage of Antibiotics, N (%)	Self-Adjustment, N (%)
Sex			
Man	734(42.3)	164(9.4)	366(21.1)
Woman	832(50.3)	232(14.0)	435(26.3)
Age			
20–24 Years	98(37.1)	35(13.3)	63(23.9)
25–39 Years	470(49.3)	133(14.0)	254(26.7)
40–54 Years	525(47.3)	128(11.5)	261(23.5)
55–69 Years	473(44.5)	100(9.4)	223(21.0)
Education			
Junior High School	46(41.4)	18(16.2)	26(23.4)
High School	574(45.4)	115(9.1)	282(22.3)
College	361(46.6)	109(14.1)	201(26.0)
University/Graduate School	555(47.9)	145(12.5)	278(24.0)
Employment			
Employed	1191(47.6)	277(11.1)	619(24.7)
Housemaker/Housewife	227(46.0)	71(14.4)	112(22.7)
Students	36(35.3)	18(17.6)	23(22.5)
Unemployed/Retired	112(38.2)	30(10.2)	47(16.0)
City Size			
Metropolitan	479(47.2)	133(13.1)	248(24.5)
Middle size City	682(47.4)	170(11.8)	337(23.4)
Small City	302(44.2)	61(8.9)	146(21.4)
Rural Area	103(40.6)	32(12.6)	70(27.6)
**Total**	**1566(46.2)**	**396(11.7)**	**801(23.6)**

Nearly half of the participants (46%) have taken antibiotics. Almost all these participants obtained antibiotics from health care institutions, and common cold was the typical reason for getting them (45.5%). While 11.7% of participants stored antibiotics at home, 23.6% of them reported having stopped or adjusted antibiotic doses by themselves. Most participants obtained their last course of antibiotics using a physician’s prescription (hospital, 84.0%; clinic, 9.5%), while 3.4% of them reported using over-the-counter (OTC) antibiotic drugs and 1.9% of them had taken left-over antibiotic drugs.

### Knowledge about antibiotics and AMR (Tables 3, 4 and 5)

**Table 3:** Knowledge about antibiotic effects.

	Antibiotics kill viruses (FALSE), N (%)			Antibiotics are effective against cold and flu (FALSE), N (%)		
	Correct	Incorrect	Don't know	Correct	Incorrect	Don't know
Sex						
Man	439(25.2)	770(44.4)	527(30.4)	452(26.0)	700(40.3)	584(33.6)
Woman	302(18.3)	817(49.4)	535(32.3)	383(23.2)	676(40.9)	595(36.0)
Age						
20–24 Years	45(17.0)	123(46.6)	96(36.4)	42(15.9)	117(44.3)	105(39.8)
25–39 Years	199(20.9)	473(49.6)	281(29.5)	248(26.0)	379(39.8)	326(34.2)
40–54 Years	252(22.7)	507(45.7)	350(31.6)	281(25.3)	435(39.2)	393(35.4)
55–69 Years	245(23.0)	484(45.5)	335(31.5)	264(24.8)	445(41.8)	355(33.4)
Education						
Junior High School	13(11.7)	48(43.2)	50(45.0)	25(22.5)	37(33.3)	49(44.1)
High School	243(19.2)	596(47.1)	426(33.7)	277(21.9)	524(41.4)	464(36.7)
College	474(24.5)	916(47.4)	542(28.1)	525(27.2)	787(40.7)	620(32.1)
University/Graduate School	27(26.5)	44(43.1)	31(30.4)	24(23.5)	43(42.2)	35(34.3)
Employment						
Employed	543(21.7)	1167(46.6)	792(31.7)	591(23.6)	1047(41.8)	864(34.5)
Housemaker/Housewife	104(21.1)	249(50.5)	140(28.4)	139(28.2)	181(36.7)	173(35.1)
Students	27(26.5)	44(43.1)	31(30.4)	24(23.5)	43(42.2)	35(34.3)
Unemployed/Retired	67(22.9)	127(43.3)	99(33.8)	81(27.6)	105(35.8)	107(36.5)
City Size						
Metropolitan	233(23.0)	456(45.0)	325(32.1)	277(27.3)	389(38.4)	348(34.3)
Midsize City	298(20.7)	695(48.3)	446(31.0)	342(23.8)	601(41.8)	496(34.5)
Small City	158(23.1)	323(47.3)	202(29.6)	159(23.3)	282(41.3)	242(35.4)
Rural	52(20.5)	113(44.5)	89(35.0)	57(22.4)	104(40.9)	93(36.6)
**Total**	**741(21.9)**	**1587(46.8)**	**1062(31.3)**	**835(24.6)**	**1376(40.6)**	**1179(34.8)**
**European Union, 2016**	**(43)**	**(46)**	**(11)**	**(56)**	**(36)**	**(8)**

**Table 4:** Knowledge about adverse effects of antibiotics.

	Unnecessary use of antibiotics makes them become ineffective (TRUE), N (%)			Taking antibiotics often has side-effects such as diarrhea (TRUE), N (%)		
	Correct	Incorrect	Don't know	Correct	Incorrect	Don't know
Sex						
Man	1207(69.5)	59(3.4)	470(27.1)	662(38.1)	230(13.2)	844(48.6)
Woman	1081(65.4)	47(2.8)	526(31.8)	653(39.5)	199(12.0)	802(48.5)
Age						
20–24 Years	163(61.7)	12(4.5)	89(33.7)	98(37.1)	37(14.0)	129(48.9)
25–39 Years	599(62.9)	41(4.3)	313(32.8)	372(39.0)	125(13.1)	456(47.8)
40–54 Years	748(67.4)	27(2.4)	334(30.1)	422(38.1)	139(12.5)	548(49.4)
55–69 Years	778(73.1)	26(2.4)	260(24.4)	423(39.8)	128(12.0)	513(48.2)
Education						
Junior High School	62(55.9)	1(0.9)	48(43.2)	43(38.7)	9(8.1)	59(53.2)
High School	778(61.5)	40(3.2)	447(35.3)	439(34.7)	141(11.1)	685(54.2)
College	1404(72.7)	64(3.3)	464(24.0)	812(42.0)	273(14.1)	847(43.8)
University/Graduate School	73(71.6)	3(2.9)	26(25.5)	41(40.2)	17(16.7)	44(43.1)
Employment						
Employed	1655(66.1)	83(3.3)	764(30.5)	938(37.5)	332(13.3)	1232(49.2)
Housemaker/Housewife	345(70.0)	13(2.6)	135(27.4)	203(41.2)	52(10.5)	238(48.3)
Students	73(71.6)	3(2.9)	26(25.5)	41(40.2)	17(16.7)	44(43.1)
Unemployed/Retired	215(73.4)	7(2.4)	71(24.2)	133(45.4)	28(9.6)	132(45.1)
City Size						
Metropolitan	692(68.2)	27(2.7)	295(29.1)	407(40.1)	124(12.2)	483(47.6)
Midsize City	982(68.2)	44(3.1)	413(28.7)	568(39.5)	178(12.4)	693(48.2)
Small City	448(65.6)	26(3.8)	209(30.6)	242(35.4)	90(13.2)	351(51.4)
Rural	166(65.4)	9(3.5)	79(31.1)	98(38.6)	37(14.6)	119(46.9)
**Total**	**2288(67.5)**	**106(3.1)**	**996(29.4)**	**1315(38.8)**	**429(12.7)**	**1646(48.6)**
**European Union, 2016**	**(84)**	**(8)**	**(8)**	**(66)**	**(14)**	**(20)**

**Table 5:** Knowledge about antimicrobial resistance.

	Heard "AMR*", N (%)	AMR*: Humans becoming immune to antibiotics (FALSE), N (%)			AMR*: Bacteria avoid being killed by antibiotics (TRUE), N (%)		
		Correct	Incorrect	Don't know	Correct	Incorrect	Don't know
Sex							
Man	759(43.7)	227(13.1)	711(41.0)	798(46.0)	839(48.3)	79(4.6)	818(47.1)
Woman	650(39.3)	104(6.3)	704(42.6)	846(51.1)	628(38.0)	72(4.4)	954(57.7)
Age							
20–24 Years	101(38.3)	28(10.6)	102(38.6)	134(50.8)	81(30.7)	25(9.5)	158(59.8)
25–39 Years	361(37.9)	81(8.5)	400(42.0)	472(49.5)	370(38.8)	57(6.0)	526(55.2)
40–54 Years	465(41.9)	103(9.3)	462(41.7)	544(49.1)	484(43.6)	42(3.8)	583(52.6)
55–69 Years	482(45.3)	119(11.2)	451(42.4)	494(46.4)	532(50.0)	27(2.5)	505(47.5)
Education							
Junior High School	26(23.4)	3(2.7)	33(29.7)	75(67.6)	32(28.8)	2(1.8)	77(69.4)
High School	423(33.4)	94(7.4)	446(35.3)	725(57.3)	447(35.3)	42(3.3)	776(61.3)
College	306(39.5)	45(5.8)	344(44.4)	385(49.7)	323(41.7)	26(3.4)	425(54.9)
University/Graduate School	627(54.1)	185(16.0)	566(48.9)	407(35.1)	639(55.2)	80(6.9)	439(37.9)
Employment							
Employed	1002(40.0)	240(9.6)	1031(41.2)	1231(49.2)	1062(42.4)	117(4.7)	1323(52.9)
Housemaker/Housewife	216(43.8)	38(7.7)	220(44.6)	235(47.7)	211(42.8)	13(2.6)	269(54.6)
Students	56(54.9)	17(16.7)	44(43.1)	41(40.2)	46(45.1)	13(12.7)	43(42.2)
Unemployed/Retired	135(46.1)	36(12.3)	120(41.0)	137(46.8)	148(50.5)	8(2.7)	137(46.8)
City Size							
Metropolitan	480(47.3)	99(9.8)	464(45.8)	451(44.5)	492(48.5)	41(4.0)	481(47.4)
Midsize City	568(39.5)	143(9.9)	561(39.0)	735(51.1)	580(40.3)	70(4.9)	789(54.8)
Small City	266(38.9)	66(9.7)	286(41.9)	331(48.5)	288(42.2)	29(4.2)	366(53.6)
Rural	95(37.4)	23(9.1)	104(40.9)	127(50.0)	107(42.1)	11(4.3)	136(53.5)
**Total**	**1409(41.6)**	**331(9.8)**	**1415(41.7)**	**1644(48.5)**	**1467(43.3)**	**151(4.5)**	**1772(52.3)**

*AMR; antimicrobial resistance

Only about 22% of participants in Japan know that antibiotics could not kill viruses. In the EU, 43% of the citizens knew that antibiotics do not kill viruses and thus twice the number of European people answered correctly. When asked if antibiotics are effective against cold and flu, only one-quarter of the participants (24.6%) provided the correct answer that they are not. Although 67.5% of the participants knew that antibiotics would not work in the future unless used properly, only about 20% of them knew what kind of diseases or pathogens they are effective for. Also, an understanding of the side effects of antibiotics was insufficient (Japan, 38.8%; EU, 66%).

Only 240 out of 3390 participants (7.1%) answered all four questions about antibiotics correctly, while 663 participants (19.6%) answered more than two questions correctly. The average number of correct answers were 1.5 out of four questions, which is lower than the response rate seen in EU in 2016 (all four correct answers, 24%; three correct answers or more, 51%; and the average number of correct answers, 2.5) [4]. Participants with better knowledge about antibacterial drugs were also more likely to use them. Participants who answered four questions correctly used antibiotics sparingly for common cold (14.5%) and flu (2.9%).

As for knowledge about AMR, only about 4 out of 10 participants had heard the term ‘AMR’. Out of the 240 participants who provided all four correct answers to questions about antimicrobials, 176 (73.3%) had heard of the term. In total, 1.6% of the participants responded to six questions correctly, including two more questions about the AMR description. Overall, based on the responses (multiple responses were allowed), the reported causes for AMR included excessive antibiotic use (46.5%), unnecessary use of antibiotics (36.8%), not completing a course of antibiotics (14.2%), and inadequate countermeasures by the medical institutions such as insufficient hand washing or inspection or no monitoring of antimicrobial resistance (6.1%). Only about 10% reported that they learned about AMR from family or friends, while 11.1% learned about it through the internet. There was a trend for decreased rate of knowing correctly the AMR definition by lower educational attainment (Table 5).

### Antibiotic information in Japan

Over half of the participants (57.5%) reported that they did not receive any such information (Table 6). The most reliable sources of information were physicians (73.5%), pharmacologists (41.6%), hospitals (21.4%), and health-related internet sites (17.1%). The most common source of antibiotic information was TV news or newspapers (25.7%) and medical doctors (19.1%). The younger group (20–39 years) were more likely to receive the information from family members or friends compared to the older group (55–69 years) (22.5% vs. 7.5%). Participants older than 55 years were more likely to receive the information from TV programs or newspaper than via the internet or other resources (32.0% vs. 7.7%).

**Table 6:** Information receipt on antibiotics, behaviour and perception among participants.

	Receiving Information, N (%)			Ask Doctors N (%)	Prefer Doctors who prescribed, N (%)
	Yes	No	Already Know		
Sex					
Man	265(15.3)	1028(59.2)	443(25.5)	181(10.4)	494(28.5)
Woman	306(18.5)	922(55.7)	426(25.8)	164(9.9)	529(32.0)
Age					
20–24 Years	40(15.2)	153(58.0)	71(26.9)	24(9.1)	67(25.4)
25–39 Years	142(14.9)	553(58.0)	258(27.1)	85(8.9)	292(30.6)
40–54 Years	179(16.1)	658(59.3)	272(24.5)	124(11.2)	355(32.0)
55–69 Years	210(19.7)	586(55.1)	268(25.2)	112(10.5)	309(29.0)
Education					
Junior High School	18(16.2)	71(64.0)	22(19.8)	12(10.8)	39(35.1)
High School	211(16.7)	781(61.7)	273(21.6)	130(10.3)	403(31.9)
College	112(14.5)	460(59.4)	202(26.1)	71(9.2)	244(31.5)
University/Graduate School	220(19.0)	586(50.6)	352(30.4)	125(10.8)	315(27.2)
Employment					
Employed	393(15.7)	1489(59.5)	620(24.8)	274(11.0)	776(31.0)
Housemaker/Housewife	108(21.9)	252(51.1)	133(27.0)	34(6.9)	147(29.8)
Students	20(19.6)	48(47.1)	34(33.3)	6(5.9)	21(20.6)
Unemployed/Retired	50(17.1)	161(54.9)	82(28.0)	31(10.6)	79(27.0)
City Size					
Metropolitan	170(16.8)	537(53.0)	307(30.3)	125(12.3)	306(30.2)
Middle size City	241(16.7)	841(58.4)	357(24.8)	136(9.5)	423(29.4)
Small City	112(16.4)	416(60.9)	155(22.7)	55(8.1)	218(31.9)
Rural Area	48(18.9)	156(61.4)	50(19.7)	29(11.4)	76(29.9)
**Total**	**571(16.8)**	**1950(57.5)**	**869(25.6)**	**345(10.2)**	**1023(30.2)**
**European Union, 2016**	**(33)**	**(65)**	**(2)**	**No Data**	**No Data**

When asked if they had changed their perception about antibiotics after receiving the information, 58.9% of the participants responded in the affirmative. While 44.5% of the participants reported that they would always consult a doctor when they needed antibiotics, 32.2% reported that they would stop self-medicating with antibiotics and 29.2% reported they would stop taking antibiotics without a prescription. Only a small number of the participants (13.2%) reported they would no longer keep left-over antibiotics for another time.

### Behaviour and perception at the time of medical visit (Table 6)

About 1 out of 10 participants (10.2%) asked for antibiotic prescriptions from their doctors at the medical visit, and 3 out of 10 participants (30.2%, 1023) believed that doctors who prescribed them for cold were good. Lastly, all participants were asked to what extent they would agree or disagree with the idea that everyone has a role ensuring that antibiotics remain effective for the next generation. Many participants totally agreed (50.4%) or tended to agree (31.5%) with the idea and thus about 80% of participants agreed with the idea. Further, about 60% of the participants who received the information had changed their views on antimicrobials.

## Discussion

In May 2015, the World Health Assembly endorsed the Global Action Plan on Antimicrobial Resistance and urged all Member States to develop relevant national action plans within two years [18]. In response to this, the National Action Plan on Antimicrobial Resistance in Japan was developed following a Ministerial Meeting on April 5th, 2016 [19]. One of the goals of this action plan is to promote public awareness and education about AMR. As a part of this project, the present survey was conducted to assess the existing knowledge, perception and behaviour about antibiotics and of Japanese people. We elucidated public issues related to AMR and barriers to the National Action Plan, pointing to the importance of wider patient education by the medical professionals in clinical practice.

Nearly half of the participants have taken antibiotics over the past year preceding the survey. Almost all antibiotics had been obtained from health care institutions.

However, the typical reason was common cold. These results are similar to the findings of studies from a Polish study [9] and many countries in Eurobarometer survey [4]. In addition, our results showed that some people had not used antimicrobials appropriately, had used leftover antibiotics at home, had discontinued or adjusted the dose by themselves. These types of uses are concerning since these could lead to greater risk of AMR. Although antibiotics can be purchased without medical prescriptions in many countries, Japanese people can obtain antibiotics only by medical prescriptions (Some were confused between antibiotics and OTC cold medications). Thus, medical professionals should have a role to better educate the public about risks associated with inappropriate use and self-discontinuation of antibiotics during treatment.

Japanese people are less likely to know that antibiotics could not kill viruses and that antibiotics are not effective against cold and flu, compared to people in the EU countries. An understanding of the side effects of antibiotics was poorer among Japanese people than those in the EU countries. It is also problematic that one third of Japanese people think antibiotic-prescribing doctors to be good. On the other hand, Swedish had the highest knowledge compared to other European participants about antibiotic ineffectiveness against viruses and common colds, and this knowledge is maintained high and has improved in 2016 from 2006 [11]. In the EU, European Antibiotic Awareness Day is organized by European Centre for Disease Prevention and Control every year to spread up-to-date knowledge regarding appropriate antibiotic use to the public. Further, Sweden developed a brochure with information regarding when people need antibiotics, why antibiotics do not help against colds and flu [11]. Therefore, more intensive dissemination of knowledge is required for the Japanese public by developing a public campaign throughout Japan and by disseminating a brochure or pamphlet to Japanese public. Since those with a better knowledge of antibiotics tended to avoid them for viral infections, it likely works to know better the facts related to these drugs.

The knowledge about AMR is not adequate among the Japanese because less than half of participants in the survey had heard the term ‘AMR’. Further, the majority had no opportunities to obtain information about antimicrobials. Although information about antibiotics and AMR has not been adequately shared to the Japanese public, the majority of them who obtained the correct information reported their wish for changes to improve the behaviour of people with regards to use of antibiotics. Italian studies demonstrated that lower educational level and unemployed status were significantly associated for not knowing the AMR definition [8, 10]. Our results in Japan also showed a trend for decreased rate of knowing correctly the AMR definition by lower educational attainment, although there was no such decreased rate among those with unemployed status in our survey. Therefore, it may be necessary to further consolidate antibiotics education among those with low educational attainment in Japan.

To prevent the development of AMR, it is necessary to share the appropriate information with the public. Although the information sharing seems still poor, such information was often obtained from newspapers and TV news, more frequently than from the doctor, suggesting that AMR has been recognized as an important problem by the Japanese media. Although medical professionals need to educate the general people about antibiotics and their biological effects, trustful media with journalism based on scientific evidence should be involved to implement the greater educational campaign for disseminating better knowledge among the public.

There are several limitations to the present study. First, this was an online survey and we compared our results with that of the Eurobarometer 445 which employed a face-to-face interview by professionals. Since our participants could not question the investigators, the data might be less accurate. In fact, on the knowledge part of the questionnaire, a higher proportion answered “Don’t know” for each question compared to the responses in the Eurobarometer 445. Second, response rate was unknown. Our survey used solicitation emails sent to a panel of internet users and we had waited until we obtained responses from over 3,126 participants (the required sample size). After sending the emails, we kept checking responses every day to confirm that we would receive responses over 3,126. At the time of day 5, we had received a total of 3,390 responses. Thus, we did not know the number of participants (denominator), who actually opened the emails and read the contents of the solicitation emails. Third, there could have been a restriction in terms of population representation, Because of the online questionnaire, the poorer people might not have internet access, but they might be the major group that consume antimicrobials with little knowledge. In fact, most of the participants (85.5%) utilized the internet almost every day. However, participants were selected with reference to the national population based on sex and age distributions in each prefecture. According to the national report in 2016, the proportion of Japanese households utilizing internet was 84.1%, and 90% of them used at least once a week (at least once a day, 75.6%; once a week, 16.4%) [20]. Fourth, the limited time frame of the survey might have led to a bias since the antimicrobial use varies across the year and the survey in different seasons might produce the different results.

Despite these limitations, our study is the first survey of a relatively large scale on this important topic. In Japan, based on the National Action Plan on Antimicrobial Resistance in 2016, government and medical professionals have strived together to tackle AMR and produced some resources for public education [21, 22]. This survey highlights the potential barriers to actions against AMR for the public. Effective public education should be an important step towards ensuring the appropriate use of antimicrobials among people.

## Conclusions

We conducted a national survey to assess the knowledge, perception, and behaviour with regards to the use of antibiotics and AMR among the Japanese public. Our results indicate that not all participants have the adequate information. Additionally, there are participants who had not taken antimicrobials appropriately, had used leftover antibiotics at home, had discontinued or adjusted the dose by themselves. Further, the majority had no opportunities to obtain information about antimicrobials, and less than half of them had heard of the term ‘AMR.’ Although information about antibiotics and AMR has not been adequately shared to the Japanese public, the majority of them who did have the correct information reported their wish for changes to improve the behaviour of people with regards to use of antibiotics. Public awareness and educational activities are necessary for developing effective countermeasures against AMR in Japan.
